# Prognostic value of neuron-specific enolase for small cell lung cancer: a systematic review and meta-analysis

**DOI:** 10.1186/s12957-020-01894-9

**Published:** 2020-05-30

**Authors:** Zhoujunyi Tian, Chaoyang Liang, Zhenrong Zhang, Huanshun Wen, Hongxiang Feng, Qianli Ma, Deruo Liu, Guangliang Qiang

**Affiliations:** Department of Thoracic Surgery, China-Japan Friendship Hospital, #2 Yinghua East Road, Chaoyang District, Beijing, 100029 China

**Keywords:** Neuron-specific enolase, Small cell lung cancer, Prognosis, Meta-analysis

## Abstract

**Background:**

Neuron-specific enolase (NSE) has become a widely used and easily attainable laboratory assay of small cell lung cancer (SCLC). However, the prognostic value of NSE for SCLC patients remains controversial. The aim of the study was to evaluate the correlation between elevated serum NSE before therapy and survival of SCLC patients.

**Methods:**

We performed a systematic review and meta-analysis. A systematic literature search was conducted in PubMed, Embase, and the Cochrane Central Register from the inception dates to December 2019. Eligible articles were included according to inclusion and exclusion criteria; then, data extraction and quality assessment were performed. The primary outcome was overall survival (OS), and the secondary endpoint was progression-free survival (PFS).

**Results:**

We identified 18 studies comprising 2981 patients. Pooled results revealed that elevated NSE was associated with worse OS (HR = 1.78, 95% CI 1.55–2.06, *p* < 0.001) and PFS (HR = 1.50, 95% CI 1.16–1.93, *p* = 0.002). In subgroup analysis, elevated NSE did not predict worse OS in patients who received only chemotherapy (HR 1.22, 95% CI 0.96–1.55, *p* = 0.10) or part of whom received surgical resection before chemotherapy and radiotherapy (HR = 2.16, 95% CI 0.82–5.69, *p* = 0.12).

**Conclusion:**

Elevated serum NSE before any therapy of SCLC patients may be a negative prognostic factor for OS and PFS. The prognostic value of NSE for OS was particularly observed in patients treated by standard management.

## Background

Lung cancer is the leading cause of cancer-related death throughout the world [1]. Small cell lung cancer (SCLC) is a deadly tumor accounting for approximately 15% of lung cancers [2]. And it is pathologically, molecularly, biologically, and clinically different from non-small cell lung cancer (NSCLC). According to whether the tumor is with distant metastasis and can be safely treated with definitive radiation doses, SCLC patients were stratified into limited disease (LD) and extent disease (ED) [3]. Twenty to 25% of patients have LD. For LD patients, chemotherapy plus thoracic radiotherapy followed by prophylactic cranial irradiation (PCI) is the standard treatment. Surgery is only available for a few early limited diseases (T1-2N0M0). The preferred treatment for ED patients is chemotherapy. Radiotherapy of the local lesion can effectively relieve symptoms [4]. Though SCLC is initially highly sensitive to chemotherapy and radiotherapy, the response rates are around 60–80% [5]. Most of patients have disease progression after treatment due to frequent resistance relapses [6, 7]. Therefore, the 5-year survival rate remains low. The median survival times are 14–20 months and 7–10 months, and 5-year survival rates are 15–25% and less than 5% in LD and ED patients, respectively [7].

Limited progress had been made in more than two decades. Encouragingly, in recent years, immunotherapy was proved to play an important role in systemic therapy of ED-SCLC [8, 9]. However, this therapy modality has not been generalized and data are limited. To increase the availability and therapeutic effect of present therapy modality and potential new treatment strategies, identification of predictive factors for survival is definitely needed. The predictive factors can help classify SCLC patients into subgroups with homogenous prognosis, which will benefit choice of treatment, studies of new therapy strategies, and comparison among studies of different medical centers.

Extent of disease and performance status (PS) have been identified as the most consistent prognostic clinical factors for survival [10–12]. Previous studies have reported various biomarkers of SCLC patients as candidates of prognostic factors, such as neuron-specific enolase (NSE), chromogranin A (cGA), neural cell adhesion molecule (NCAM), caspase cleaved cytokeratin 19 (CYFRA21.1), tissue polypeptide antigen (TPA), carcinoembryonic antigen (CEA), lactate dehydrogenase (LDH), and neutrophil-to-lymphocyte ratio (NLR) [13, 14].

NSE, also known as enolase-γ, is a neuro- and neuroendocrine-specific isoenzyme of enolase, which is a key enzyme in aerobic glycolysis. NSE is localized to neurons and neuroendocrine cells of the amine precursor uptake and decarboxylation (APUD) series [15]. It is found in several neuroendocrine origin or neuronal tumors such as SCLC and neuroblastoma [13, 16]. Also, it is expressed in normal tissue, for example, neuroendocrine tissues, erythrocytes, smooth muscle cells, plasma cells, and platelets [17]. Early in the 1980s, researchers established cell lines from SCLC and demonstrated expression of NSE [18, 19]. The levels of NSE in SCLC cell lines were significantly higher than those derived from other types of lung cancer [15]. Serum NSE level is reported to be frequently elevated in SCLC at the time of diagnosis, reduced after remission, and rebounded after relapse [20–22]. It made NSE a very important tumor marker of SCLC. Nowadays, NSE has become a widely used and easily attainable laboratory assay of SCLC patients. However, the prognostic value of NSE in SCLC patients remains controversial according to results from many researches. This study is to evaluate the prognostic significance of serum NSE in SCLC patients through systematic review with meta-analysis of the published literature.

## Material and methods

### Search strategy

The PubMed, Cochrane Library, and Embase databases were searched from the inception dates to December 2019, to identify researches that meet the inclusion criteria of this review. There was no language restriction. The search terms were based on keywords, including “small cell lung cancer,” “neuron-specific enolase,” and “prognosis.” The detailed search strategies are provided in the supplementary data. The reference lists of every article were checked for relevant articles. The protocol of this meta-analysis was open on PROSPERO, the International Prospective Register of Systematic Reviews (CRD42020160753).

### Inclusion and exclusion criteria

Inclusion criteria for selecting were as follows: (i) the research subjects are SCLC patients confirmed by pathological or cytological examination; (ii) serum NSE was measured at least once before any therapy; (iii) a cutoff value of serum NSE was defined to dichotomize the level as “normal” or “high/abnormal/elevated” value; (iv) sufficient information to allow extracting directly or calculating the correlation of NSE with overall survival (OS) and/or progression-free survival (PFS), expressed by individual hazard ratio (HR) and its variance; and (v) if sample was overlapped in different published studies, only the most informative and recent research was included. The exclusion criteria were as follows: (i) duplicated articles and (ii) basic research, abstracts, letters, case reports, reviews, and other informally published forms.

### Data extraction and quality assessment

Literature screening and identification were performed by two independent reviewers. If disagreement occurred, two authors discussed and arrived at consensus with a third author. The following information from each study was recorded: first author, publication year, study design, source of patient, sample sizes, age of patients, gender of patients, extent of disease, cutoff value of the serum NSE, treatment protocol, follow-ups, and outcome data (OS, PFS, and corresponding effect sizes). If data from any of the above categories were not given in the primary studies, items were treated as “not applicable.”

Quality assessment of the included studies was conducted using the Newcastle-Ottawa Scale (NOS). The NOS consists of three parts: selection (0–4 points), comparability (0–2 points), and outcome assessment (0–3 points). Two independent reviewers assessed the risk of bias of each study. Disagreement was resolved by discussion or consultation with an independent third adjudicator.

### Statistical analysis

The primary endpoint was OS, which was defined as the interval from treatment until time of death from any cause. The secondary endpoint was PFS, which was defined as the interval from treatment until time of first progression or death from any cause. The effect sizes namely HR and 95% CI of the dichotomous variable (“normal” or “high” level of serum NSE) were obtained directly from each literature. If the HRs were not available, we calculated the effect sizes using other survival data according to the methods illustrated by Tierney et al. [23]. A HR > 1 indicated a worse prognosis in SCLC patients with elevated level of NSE before treatment. Cochran’s *Q* test and Higgins’ *I*^2^ statistic were conducted to assess the heterogeneity of the included researches. If *I*^2^ was > 50%, a substantial level of heterogeneity may exist among these researches, in which case a random effect model was used. Otherwise, a fixed effect model was used. In cases of substantial heterogeneity, we performed subgroup analysis for OS according to study design, ethnicity, cutoff value of NSE, and treatment protocol and conducted meta-regression to explore and explain the probable source of heterogeneity. We also conducted sensitivity analysis to assess the influence of individual study to the overall effect size estimate. Publication bias was assessed by Egger’s linear regression and Begg’s funnel plot [24]. A two-sided *p* value < 0.05 was considered statistically significant. Data combination and statistical analyses were performed using the Review Manager (RevMan) software (V.5.3, Cochrane Collaboration) and Stata/SE version 14.0 for Windows (StataCorp, College Station, TX, USA).

## Result

### Study characteristics

**Fig. 1:**
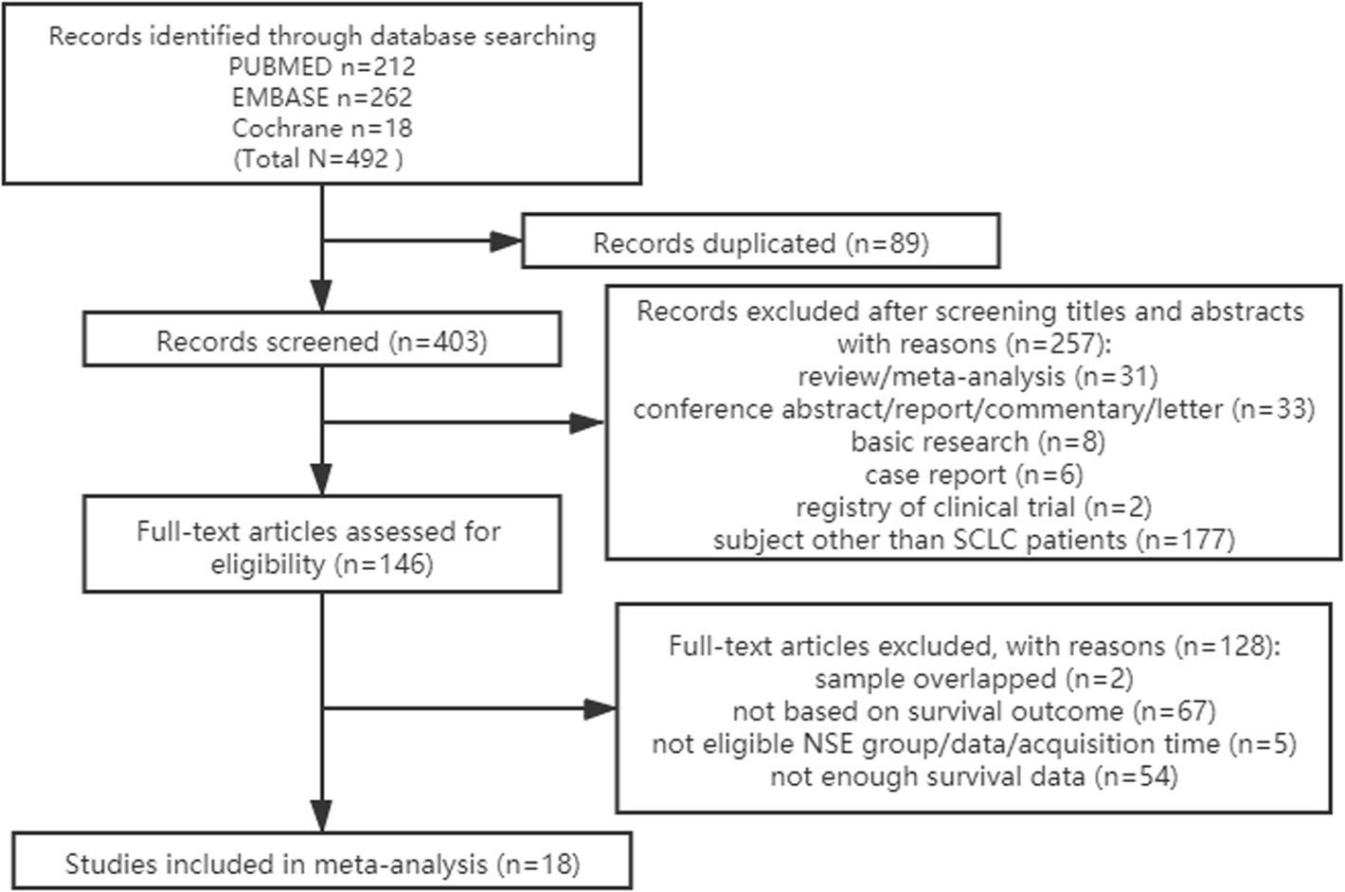
Flow diagram for identification of eligible studies

The process for identification of eligible articles is shown in Fig. 1. A total of 492 items were identified (212 from PubMed, 262 from Embase, and 18 from the Cochrane library). After meticulous screening and inspection of the articles, finally, 18 articles were included in our final analysis [21, 25–41].

Table 1 summarizes the characteristics of the included studies. The total number of patients in our systematic review and meta-analysis was 2981. All studies were published between 1991 and 2019. Sixteen of all 18 studies were retrospective studies. In the rest two studies, sample of Bremnes et al.’s study [31] was comprised of patients included in another prospective multicenter study [42], while Liu et al.’s study [39] was a prospective single-armed study. Treatment protocol “C” refers to that patients received only chemotherapy, “C,R” refers to that patients received chemotherapy with or without radiotherapy, and “S,C,R” refers to that part of patients received surgical resection before chemotherapy and radiotherapy. Effect sizes of correlation between serum level of NSE and OS were available in 16 studies, while those between serum level of NSE and PFS were available in only 4 studies. NOS scores of all studies were ≥ 6 stars.

**Table 1:** Main characteristics of studies included in the meta-analysis

Study	Year	Study design	Ethnicity	*N*	Percentage of LD-SCLC	Percentage of male patients	Age (median)	Age (mean)	Age (range)	Cutoff of NSE (ng/ml)	Treatment	Outcome	NOS
van der Gaast A [25]	1991	Retrospective	Caucasian	70	41.4%	77.1%	63		36–75	12.5	C	OS	7
Johnson PW [26]	1993	Retrospective	Caucasian	154	29.2%	65.0%	63		34–77	25	C,R	OS	7
Fischer JR [27]	1997	Retrospective	Caucasian	52	63.5%	75.0%		58	36–75	30	C,R	OS	6
Shibayama T [28]	2001	Retrospective	Asian	114	59.1%	86.8%	65		29–82	7.5	C,R	OS	6
Jin B [29]	2001	Retrospective	Asian	144	NA	85.4%	62.12		36–86	12.5	C	OS	6
Jean-Louis Pujol [30]	2003	Retrospective	Caucasian	148	39.2%	93.9%	63	61	42–82	17	C,R	OS	7
Bremnes RM [31]	2003	Prospective	Caucasian	436	49.1%	64.2%	64		39–76	13	C,R	OS	7
Ando S [32]	2004	Retrospective	Asian	57	64.9%	84.2%		66	48–78	10.5	S,C,R	OS	6
Xue F [33]	2011	Retrospective	Asian	57	52.6%	68.4%	52.5		29–70	15.2	C,R	OS	6
Zhu H [34]	2015	Retrospective	Asian	281	55.5%	80.4%	57		25–82	18	S,C,R	OS	7
Huang Z [21]	2016	Retrospective	Asian	122	44.3%	75.4%	NA	NA	NA	17	C,R	PFS	6
Wojcik E [35]	2016	Retrospective	Caucasian	63	65.1%	60.3%	59		32–76	35	C,R	OS	6
Jiang X [36]	2017	Retrospective	Asian	107	39.3%	78.5%	63		58.5–68	17	C,R	OS	7
Pan H [37]	2017	Retrospective	Asian	275	NA	87.0%		62.59		81.4	C,R	OS	7
Zhou M [38]	2017	Retrospective	Asian	523	26.8%	79.3%	59		27–87		C,R	OS	7
Liu X [39]	2017	Prospective	Asian	136	43.4%	64.7%		53.3		50.324	C,R	OS, PFS	6
Zhang C [40]	2018	Retrospective	Asian	160	23.8%	80.6%	59		23–83		C	PFS	7
Fan S [41]	2019	Retrospective	Asian	82	NA	81.7%	60		28–82	16.3	C,R	OS, PFS	7

*Abbreviations*: *LD-SCLC* limited disease small cell lung cancer, *C* chemotherapy, *R* radiotherapy, *S* surgery, *OS* overall survival, *PFS* progression-free survival, *NA* not available

### NSE and OS in SCLC

**Fig. 2:**
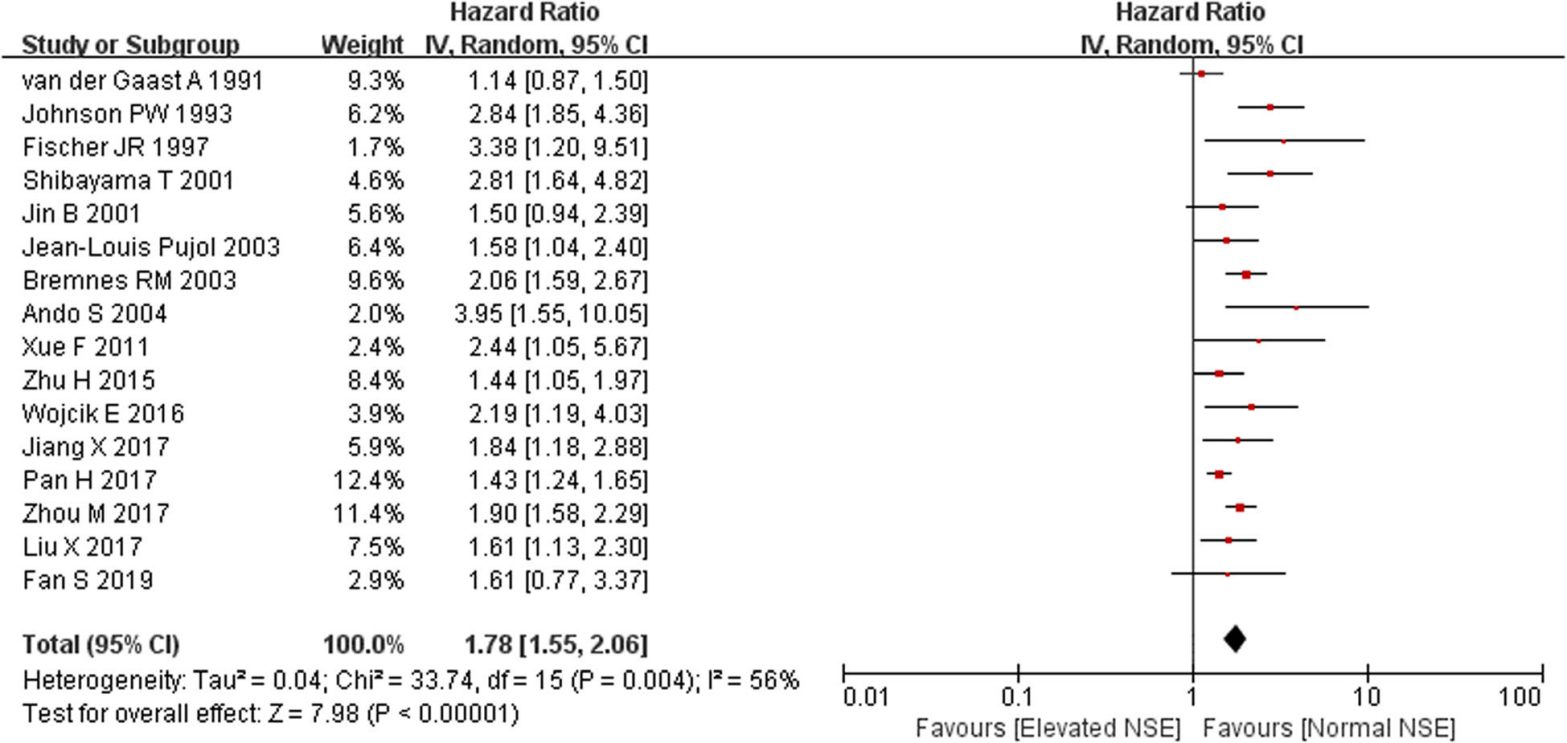
Forest plots of hazard ratios (HRs) for overall survival according to pretreatment serum NSE in SCLC patients

Meta-analysis was conducted on 16 studies with effect size data of OS. However, the heterogeneity was moderate (*I*^2^ = 56%, *Q* = 33.74, *p* = 0.004); therefore, pooled estimates were weighted and combined using a random effect model. The results indicated that elevated NSE predicted a poorer OS for SCLC patients, with the combined HR of 1.78 (95% CI 1.55–2.06, *p* < 0.001; Fig. 2).

In the subgroup analysis for OS according to treatment protocol, elevated NSE did not have significant prognostic value of OS for SCLC patients treated by only chemotherapy in 2 studies [25, 29] with combined HR of 1.22 (95% CI 0.96–1.55, *p* = 0.10; *I*^2^ = 0%, *p* = 0.32). In addition, elevated NSE did not have significant prognostic value of OS for SCLC patients part of whom received surgical resection before chemotherapy and radiotherapy in 2 studies [32, 34] with combined HR of 2.16 (95% CI 0.82–5.69, *p* = 0.12; *I*^2^ = 75%), while the combined HR of the remaining 13 studies indicated that elevated NSE may predict worse OS (HR 1.90, 95% CI 1.63–2.20, *p* < 0.001; *I*^2^ = 48%, *p* = 0.03) in SCLC patients who received chemotherapy with or without radiotherapy.

The meta-regression technique performed using the model weighted by the inverse of the variance was also used to explore the source heterogeneity. We investigated study design, ethnicity, cutoff value of NSE, and treatment as probable sources of heterogeneity, and there were no significant factors identified (coefficient 0.96, 95% CI 0.45–1.46; coefficient 0.96, 95% CI 0.57–1.36; coefficient 1.01, 95% CI 0.63–1.39; coefficient 0.89, 95% CI 0.61–1.18; respectively).

**Fig. 3:**
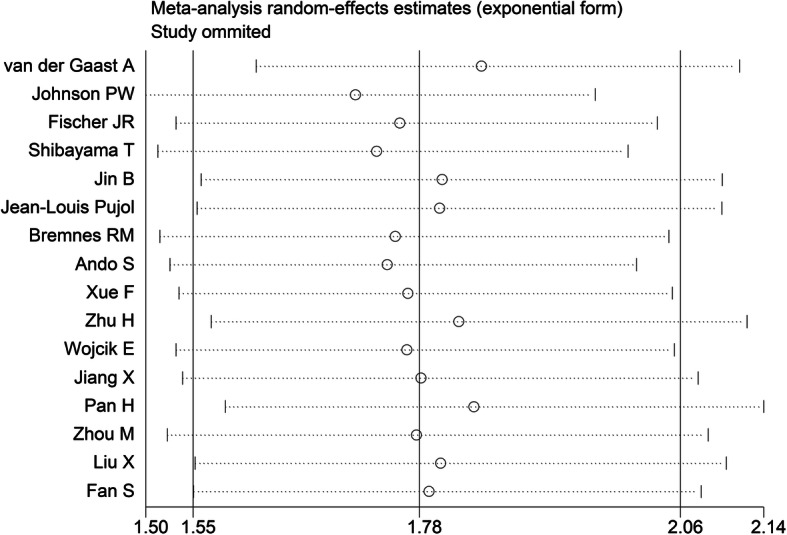
Influence analysis plots of combined effect sizes when each study was excluded

We also performed sensitivity analysis to investigate the influence of each study on the overall meta-analysis estimate by calculating the pooled HRs with successive exclusion of one study. None of these studies had a significant interference to combined HRs than the other studies (Fig. 3).

### NSE and PFS in SCLC

**Fig. 4:**
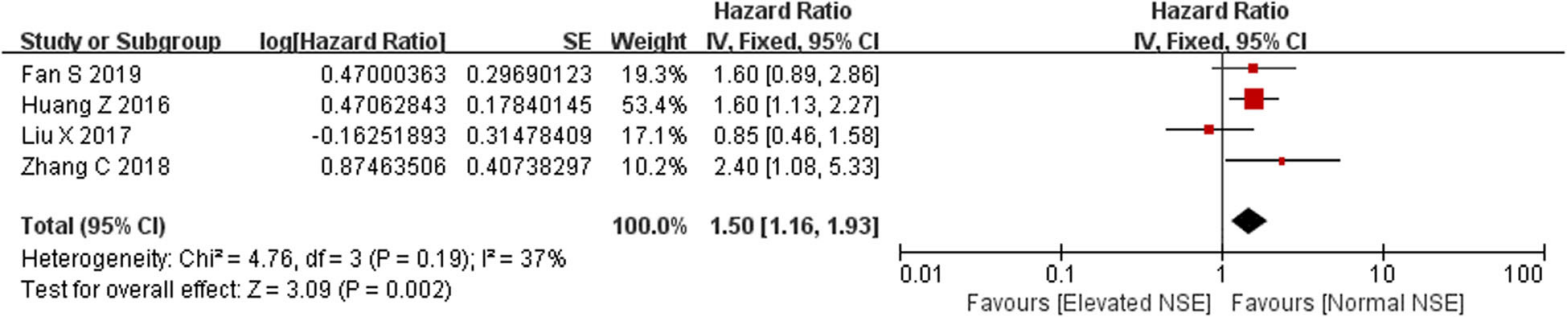
Forest plots of hazard ratios (HRs) for progression-free survival according to pretreatment serum NSE in SCLC patients

Meta-analysis was conducted on 4 studies with effect size data of PFS. The heterogeneity was mild (*I*^2^ = 37.0%, *Q* = 4.76, *p* = 0.19); therefore, pooled estimates were weighted and combined using a fixed effect model. The results indicated that elevated NSE predicted a poorer PFS for SCLC patients, with the combined HR of 1.50 (95% CI 1.16–1.93, *p* = 0.002; Fig. 4).

### Publication bias

Egger’s linear regression test and Begg’s funnel plot were performed to evaluate publication bias. Publication bias was detected for OS by Egger’s test (*p* = 0.04), but not detected by Begg’s test (*p* = 0.10).

## Discussion

In this study, we investigated the prognostic value of serum NSE before therapy in SCLC patients using a systemic review and meta-analysis approach. The combined HR from 16 of the included studies indicated that elevated serum NSE may predict worse OS (HR 1.78, 95% CI 1.55–2.06, *p* < 0.001). In addition, the combined HR from 4 of the included studies revealed that elevated serum NSE may also predict poorer PFS (HR 1.50, 95% CI 1.16–1.93, *p* = 0.002). Serum NSE could be secreted by SCLC tumor cells, and its level is related to tumor mass extension [22, 43]. Thus, serum NSE level is associated with the tumor burden. Liu’s team silenced NSE in SCLC cell lines using a loss-of-function approach and found that the knockdown of NSE suppressed proliferation, colony formation, and migration of SCLC cells, compared to those of the control group. Also, the silencing of NSE led to the downregulation of metastasis promoter gene vascular endothelial growth factor (VEGF) and upregulation of metastasis suppressor genes NM23 and E-cadherin [44]. These experimental results may support that the elevated NSE of SCLC patients is associated with unfavorable outcome.

Subgroup analysis was conducted yet failed to find the source of heterogeneity. In subgroup analysis for OS according to treatment protocol, the prognostic value of serum NSE for OS was only observed in SCLC patients treated by chemotherapy with or without radiotherapy. Our interpretations for the results of the subgroup analysis are as follows. First, the number of studies included in the other two subgroups was limited, which may cause limited sample size and restrict the statistic power. Secondly, chemotherapy and radiotherapy are standard treatment modality for most of SCLC patients [4.] Only a very limited part (< 5%) of patients had opportunity to receive surgical resection [4]. The relatively high proportion of surgical management in the 2 studies [32, 34] of the subgroup S,C,R may impair the representativeness of sample. Likewise, patients in the 2 studies [25, 29] of the subgroup C received only chemotherapy, but they did not detail the reason. Thirdly, in clinical practice, the prognostic value of NSE may only be expressed on condition that patients received standard management.

Moderate heterogeneity for the outcomes of interest existed in this meta-analysis. Although we investigated potential sources of heterogeneity, they were not identified in meta-regression analysis. In addition, the result of sensitivity analysis implied that each individual study did not have significant influence to overall combined HR.

**Fig. 5:**
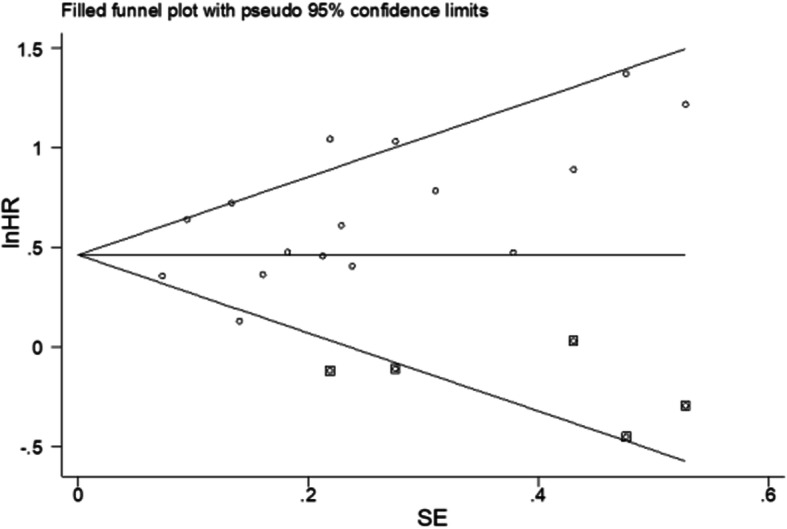
Funnel plot after trim and fill method. The small circles on the plot refer to the 16 studies included in the meta-analysis for correlation between serum NSE and OS, while the small diamonds on the plot refer to the 5 hypothetic studies needed to trim the plot to make it symmetric

Limited by the rather low incidence of SCLC, the existing studies were almost retrospective. Due to the relatively limited quality of studies, only officially published studies can be included, though we performed the literature search as thoroughly as possible to minimize publication bias. However, Egger’s test and the asymmetric funnel plot implied that the publication bias cannot be excluded. In sensitivity analysis, we also performed a trim and fill method to evaluate the stability of combined HR. Figure 5 reveals that 5 studies were needed to counteract the publication bias. However, when the 5 studies were added, the combined HR did not change significantly (HR 1.61, 95% CI 1.38–1.87, *p* < 0.001). It indicated that the publication bias may not affect the result.

Watine [45] published a systematic review in 1999 and tried to establish laboratory parameters including NSE, to give the pretreatment prognostic information in small cell lung cancer (SCLC) patients. However, the prognostic value of NSE was not demonstrated at that time. A meta-analysis of similar theme was published in 2013 [46]. But this meta-analysis only assessed the impact of NSE on OS. What is more, since then, there were still original researches with negative results published [41]. The prognostic value of NSE still remains controversial. Our meta-analysis was not only an update. We also performed subgroup analysis to evaluate the condition of applying NSE. Meta-regression was also adopted to explore the source of heterogeneity. Sensitivity analysis was performed to evaluate the stability of result. Furthermore, the trim and fill method was used to evaluate the influence of publication bias.

Several potential limitations of our study should be considered when interpreting the results. Firstly, most of the included studies were retrospective, which were more susceptible to some biases. Secondly, different thresholds of serum NSE level and detection technologies were adopted in these studies. Though NSE in these studies was dichotomized according to the threshold value, the bias was non-negligible. Third, we combined the HRs from univariate analysis using Cox proportional hazard model of each study, which were unadjusted for other factors, because HR from multivariate analysis was adjusted with different kinds and number of factors and regressed by different models in individual study, which may sometimes be excluded from model and cause more bias. Finally, there was a statistically significant heterogeneity in the included studies and publication bias cannot be excluded.

## Conclusion

Elevated serum NSE before any therapy of SCLC patients may be a negative prognostic factor for OS and PFS. The prognostic value of NSE for OS was particularly observed in patients treated by standard management. Further trials of high evidence level are needed to sustain the conclusion.

## Supplementary information

**Additional file 1:.** Supplement data-search strategy

## Data Availability

The data used and analyzed in the current study are available from the corresponding author upon reasonable request.

## Abbreviations

SCLC: Small cell lung cancer
NSCLC: Non-small cell lung cancer
LD: Limited disease
ED: Extent disease
PCI: Prophylactic cranial irradiation
PS: Performance status
NSE: Neuron-specific enolase
cGA: Chromogranin A
NCAM: Neural cell adhesion molecule
CYFRA21.1: Caspase cleaved cytokeratin 19
TPA: Tissue polypeptide antigen
CEA: Carcinoembryonic antigen
LDH: Lactate dehydrogenase
NLR: Neutrophil-to-lymphocyte ratio
OS: Overall survival
PFS: Progression-free survival
HR: Hazard ratio
CI: Confidence interval
NOS: Newcastle-Ottawa Scale
APUD: Amine precursor uptake and decarboxylation
VEGF: Vascular endothelial growth factor
