# Supplementary material for: Prognostic value of neuron-specific enolase for small cell lung cancer: a systematic review and meta-analysis
Source: World J Surg Oncol. 2020 May 30;18:116. doi: 10.1186/s12957-020-01894-9 (PMC7261386; doi:10.1186/s12957-020-01894-9)
Supplement: Supplementary file 1 — Additional file 1:. Supplement data-search strategy [file 12957_2020_1894_MOESM1_ESM.pdf]

The search strategy of our review:

PUBMED:

((((("Small Cell Lung Carcinoma"[Mesh]) OR Small Cell Lung Cancer[Title/Abstract]) OR Oat Cell Lung Cancer[Title/Abstract]) OR Small Cell Cancer Of The Lung[Title/Abstract]) OR Carcinoma, Small Cell Lung[Title/Abstract]) OR Oat Cell Carcinoma of Lung[Title/Abstract] AND (("Phosphopyruvate Hydratase"[Mesh]) OR (((((((((((((((((((((((((((((((Hydratase, Phosphopyruvate[Title/Abstract]) OR 2-Phospho-D-Glycerate Hydrolase[Title/Abstract]) OR 2-Phospho-D-Glycerate Hydrolase[Title/Abstract]) OR Enolase[Title/Abstract]) OR 2-Phospho-D-Glycerate Hydro-Lyase[Title/Abstract]) OR 2 Phospho D Glycerate Hydro Lyase[Title/Abstract]) OR Hydro-Lyase, 2-Phospho-D-Glycerate[Title/Abstract]) OR 2-Phosphoglycerate Dehydratase[Title/Abstract]) OR 2 Phosphoglycerate Dehydratase[Title/Abstract]) OR Dehydratase, 2-Phosphoglycerate[Title/Abstract]) OR gamma-Enolase[Title/Abstract]) OR gamma Enolase[Title/Abstract]) OR Nervous System-Specific Enolase[Title/Abstract]) OR Enolase, Nervous System-Specific[Title/Abstract]) OR Nervous System Specific Enolase[Title/Abstract]) OR System-Specific Enolase, Nervous[Title/Abstract]) OR Neuron-Specific Enolase[Title/Abstract]) OR Enolase, Neuron-Specific[Title/Abstract]) OR Neuron Specific Enolase[Title/Abstract]) OR Enolase 2[Title/Abstract]) OR alpha-Enolase[Title/Abstract]) OR alpha Enolase[Title/Abstract]) OR Non-Neuronal Enolase[Title/Abstract]) OR Enolase, Non-Neuronal[Title/Abstract]) OR Non Neuronal Enolase[Title/Abstract]) OR beta-Enolase[Title/Abstract]) OR beta Enolase[Title/Abstract]) OR Muscle-Specific Enolase[Title/Abstract]) OR Enolase, Muscle-Specific[Title/Abstract]) OR Muscle Specific Enolase[Title/Abstract]) OR Enolase 3[Title/Abstract]))) AND (("Prognosis"[Mesh]) OR (((((Prognoses[Title/Abstract]) OR Prognostic Factors[Title/Abstract]) OR Factor, Prognostic[Title/Abstract]) OR Factors, Prognostic[Title/Abstract]) OR Prognostic Factor[Title/Abstract]))

Cochrane Library:

1. MeSH descriptor: [Small Cell Lung Carcinoma] explode all trees
2. Carcinoma, Small Cell or Carcinomas, Small Cell or Small Cell Carcinomas or Oat Cell Carcinoma or Small Cell Carcinoma or Carcinoma*, Oat Cell or Oat Cell Carcinoma*
3. #1 or #2
4. MeSH descriptor: [Phosphopyruvate Hydratase] explode all trees
5. Hydratase, Phosphopyruvate or 2*Phospho*D*Glycerate Hydrolase or 2 Phospho D Glycerate Hydrolase or Enolase or 2*Phospho*D*Glycerate Hydro*Lyase or 2 Phospho D Glycerate Hydro Lyase or Hydro*Lyase, 2*Phospho*D*Glycerate or 2*Phosphoglycerate Dehydratase or 2 Phosphoglycerate Dehydratase or Dehydratase, 2*Phosphoglycerate or gamma*Enolase or gamma Enolase or Nervous System*Specific Enolase or Enolase, Nervous System*Specific or Nervous System Specific Enolase or System*Specific Enolase, Nervous or Neuron*Specific Enolase or Enolase, Neuron*Specific or Neuron Specific Enolase or Enolase 2 or alpha*Enolase or alpha Enolase or Non*Neuronal Enolase or Enolase, Non*Neuronal or Non Neuronal Enolase or beta*Enolase or beta Enolase or Muscle*Specific Enolase or Enolase, Muscle*Specific or Muscle Specific Enolase or Enolase 3
6. #4 or #5
7. #3 and #6

Embase:

1. 'small cell lung cancer'/exp
2. 'carcinoma, small cell':ab,ti OR 'carcinomas, small cell':ab,ti OR 'small cell carcinomas':ab,ti OR 'oat cell carcinoma':ab,ti OR 'small cell carcinoma':ab,ti OR 'carcinoma, oat cell':ab,ti OR 'carcinomas, oat cell':ab,ti OR 'oat cell carcinomas':ab,ti
3. #1 OR #2
4. 'enolase'/exp
5. 'phosphopyruvate hydratase' OR 'hydratase, phosphopyruvate' OR '2-phospho-d-glycerate hydrolase' OR '2 phospho d glycerate hydrolase' OR '2-phospho-d-glycerate hydro-lyase' OR '2 phospho d glycerate hydro lyase' OR 'hydro-lyase, 2-phospho-d-glycerate' OR '2-phosphoglycerate dehydratase' OR '2 phosphoglycerate dehydratase' OR 'dehydratase, 2-phosphoglycerate' OR 'gamma-enolase' OR 'gamma enolase' OR 'nervous system-specific enolase' OR 'enolase, nervous system-specific' OR 'nervous system specific enolase' OR 'system-specific enolase, nervous' OR 'neuron-specific enolase' OR 'enolase, neuron-specific' OR 'neuron specific enolase' OR 'enolase 2' OR 'alpha-enolase' OR 'alpha enolase' OR 'non-neuronal enolase' OR 'enolase, non-neuronal' OR 'non neuronal enolase' OR 'beta-enolase' OR 'beta enolase' OR 'muscle-specific enolase' OR 'enolase, muscle-specific' OR 'muscle specific enolase' OR 'enolase 3':ab,ti
6. #4 OR #5
7. 'prognosis'/exp
8. 'prognoses':ab,ti OR 'prognostic factors':ab,ti OR 'factor, prognostic':ab,ti OR 'factors, prognostic':ab,ti OR 'prognostic factor':ab,ti OR 'prognostic':ab,ti
9. #7 OR #8
10. #3 AND #6 AND #9
